# TRPC3-Nox2 axis mediates nutritional deficiency-induced cardiomyocyte atrophy

**DOI:** 10.1038/s41598-019-46252-2

**Published:** 2019-07-05

**Authors:** Suhaini Binti Sudi, Tomohiro Tanaka, Sayaka Oda, Kazuhiro Nishiyama, Akiyuki Nishimura, Caroline Sunggip, Supachoke Mangmool, Takuro Numaga-Tomita, Motohiro Nishida

**Affiliations:** 1 National Institute for Physiological Sciences (NIPS), National Institutes of Natural Sciences, Okazaki, 444-8787 Japan; 2 Exploratory Research Center on Life and Living Systems (ExCELLS), National Institutes of Natural Sciences, Okazaki, 444-8787 Japan; 3 Faculty of Medicine and Health Sciences, University Malaysia Sabah, Kota Kinabalu, 88400 Malaysia; 4 Center for Novel Science Initiatives (CNSI), National Institutes of Natural Sciences, Tokyo, 105-0001 Japan; 5 SOKENDAI (School of Life Science, The Graduate University for Advanced Studies), Okazaki, 444-8787 Japan; 6 Graduate School of Pharmaceutical Sciences, Kyushu University, Fukuoka, 812-8582 Japan; 7 Faculty of Pharmacy, Mahidol University, Bangkok, 10400 Thailand

**Keywords:** Heart failure, Heart failure

## Abstract

Myocardial atrophy, characterized by the decreases in size and contractility of cardiomyocytes, is caused by severe malnutrition and/or mechanical unloading. Extracellular adenosine 5′-triphosphate (ATP), known as a danger signal, is recognized to negatively regulate cell volume. However, it is obscure whether extracellular ATP contributes to cardiomyocyte atrophy. Here, we report that ATP induces atrophy of neonatal rat cardiomyocytes (NRCMs) without cell death through P2Y_2_ receptors. ATP led to overproduction of reactive oxygen species (ROS) through increased amount of NADPH oxidase (Nox) 2 proteins, due to increased physical interaction between Nox2 and canonical transient receptor potential 3 (TRPC3). This ATP-mediated formation of TRPC3-Nox2 complex was also pathophysiologically involved in nutritional deficiency-induced NRCM atrophy. Strikingly, knockdown of either TRPC3 or Nox2 suppressed nutritional deficiency-induced ATP release, as well as ROS production and NRCM atrophy. Taken together, we propose that TRPC3-Nox2 axis, activated by extracellular ATP, is the key component that mediates nutritional deficiency-induced cardiomyocyte atrophy.

## Introduction

The heart is capable of remodeling in response to workload by modulating protein synthesis and degradation. Cardiac atrophy is defined as the reduction in myocardial mass induced by various factors, such as nutritional deficiency, disuse, and muscle loss in chronic inflammatory diseases including cancer-induced cachexia^1^.

The hallmark of cardiac atrophy is the increase in protein degradation that resulted in activation of ubiquitin proteasome system (UPS)-mediated proteolysis that consequently activates autophagy for protein clearance in cardiomyocytes^2,3^. Starvation, mechanical unloading and cancer promote protein degradations to provide substrates for gluconeogenesis as well as energy sources required for cellular homeostasis. Starvation-induced cardiac atrophy activates adenosine monophosphate (AMP)-activated protein kinase (AMPK), which leads to autophagy and inhibition of protein synthesis through suppression of mammalian target of rapamycin (mTOR) signaling pathway^4^. In multiple types of muscle atrophy, protein synthesis is attenuated with the upregulation of ubiquitin ligases atrogin-1/MAFbx and muscle ring finger-1 (MuRF1), and several autophagy markers including LC3-phosphatidylethanolamine conjugate (LC3-II), cathepsin L and beclin^5,6^. These cellular responses are mediated by FoxO family of transcription factors, such as FoxO1 and FoxO3^5,6^. However, key regulator(s) promoting atrophy of cardiac muscle remain obscure.

Sustained release of adenosine 5′-triphosphate (ATP) occurs in response to physiological and pathophysiological conditions, such as environmental stress, pathogenic bacterial invasion and inflammation in an immune system^7^. Meanwhile, ATP is also released during osmotic swelling, and it has been demonstrated that extracellular ATP is required for recovery from swelling^8^. In the cardiovascular system, extracellular ATP is an important vasoactive signaling molecule and elicits potent effects on vascular cells to regulate vessel tones^9–11^. In the heart and skeletal muscles, ATP stimulates purinergic P2 receptors (P2X and P2Y) and initiates signaling cascades such as increase in intracellular calcium (Ca^2+^) level, activation of phospholipases and elevation of cardiac or skeletal muscle contractility, otherwise rapidly metabolized by ecto-enzymes^12^.

Among P2 receptors, P2Y_2_ receptor is highly expressed in cardiomyocytes, and is mainly coupled to G_q_ type heterotrimeric GTP-binding proteins. Upon stimulation by ATP or UTP, P2Y_2_ receptor activates phospholipase C that hydrolyzes plasma membrane phosphatidylinositol-4,5-bisphosphate, generating two second messenger molecules; inositol triphosphate (IP_3_) and diacylglycerol^13^. IP_3_ activates IP_3_ receptor, localized at smooth endoplasmic reticulum, and stimulates the release of stored Ca^2+^ ^14,15^. Despite being a potent activator of Ca^2+^-dependent nuclear factor of activated T cells (NFAT), ATP does not induce cardiomyocyte hypertrophy^16,17^. This could be partially explained by our previous study, where ATP activates endothelial nitric oxide synthase which negatively regulates NFAT-dependent hypertrophic responses in cardiomyocytes^18^. However, it remains to be elucidated whether and how ATP induces cardiomyocyte atrophy.

We previously reported that TRPC3 and Nox2 form a complex when cardiac atrophy was induced by doxorubicin, an anticancer drug^19^. Importantly, TRPC3-Nox2 complex stabilizes and prevents Nox2 from proteasome-dependent degradation, whereby promoting reactive oxygen species (ROS) production and its downstream maladaptive signaling, which is induced by mechanical stretch during diastolic filling in cardiomyocytes^20^. Furthermore, several findings have suggested that ROS mediate protein degradation and atrophy in muscle during starvation and oxidative stress^21–23^. Here, we show that high level of extracellular ATP, which is induced by nutritional deficiency, mediates atrophy in neonatal rat cardiomyocytes (NRCMs), via formation of TRPC3-Nox2 complex.

## Results

### Extracellular ATP induces atrophy in NRCMs without cell death

**Figure 1:**
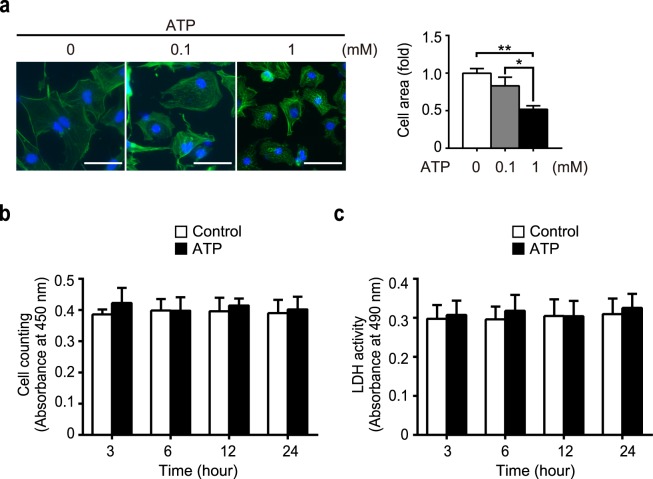
Extracellular ATP induces cardiomyocyte atrophy without cell death. (**a**) Representative images of phalloidin (green) and DAPI (blue) on NRCMs stimulated with extracellular ATP (0.1 mM, 1 mM) for 24 h (left). Scale bars, 50 μm. Fold increase of averaged cell area (right, n = 3). (**b**,**c**) Effects of ATP on cell viability, measured with cell counting assay (**b**) and LDH assay for cytotoxicity (**c**). Cells were stimulated with 1 mM ATP up to 24 h. n = 3. *P < 0.05; **P < 0.01 (one-way ANOVA; Tukey’s *post hoc* test).

Our previous study have shown that ATP negatively regulates hypertrophic signaling via TRPC3/6 channel in cardiomyocytes^18^. This finding led us to investigate further the potential of ATP as an anti-hypertrophic molecule in cardiomyocytes. We first examined the effect of low (0.1 mM) and high (1 mM) concentrations of ATP on NRCMs. Treatment with ATP decreased cell size in a dose-dependent manner, and remarkably, high ATP caused 50% reduction in cell size compared to non-treated control NRCMs (Fig. 1a). To investigate whether this cell shrinkage is due to cytotoxicity that eventually leads to cell death, we used a lactate dehydrogenase (LDH) assay and quantified the number of cells, measuring the viability of high ATP-treated NRCMs. There were no significant change of cell viability by ATP treatment (Fig. 1b,c), suggesting that ATP-induced decrease in cell size is not due to cytotoxicity but an atrophic remodeling of cardiomyocytes.

### ATP treatment evokes ROS production via upregulation of Nox2 protein abundance

**Figure 2:**
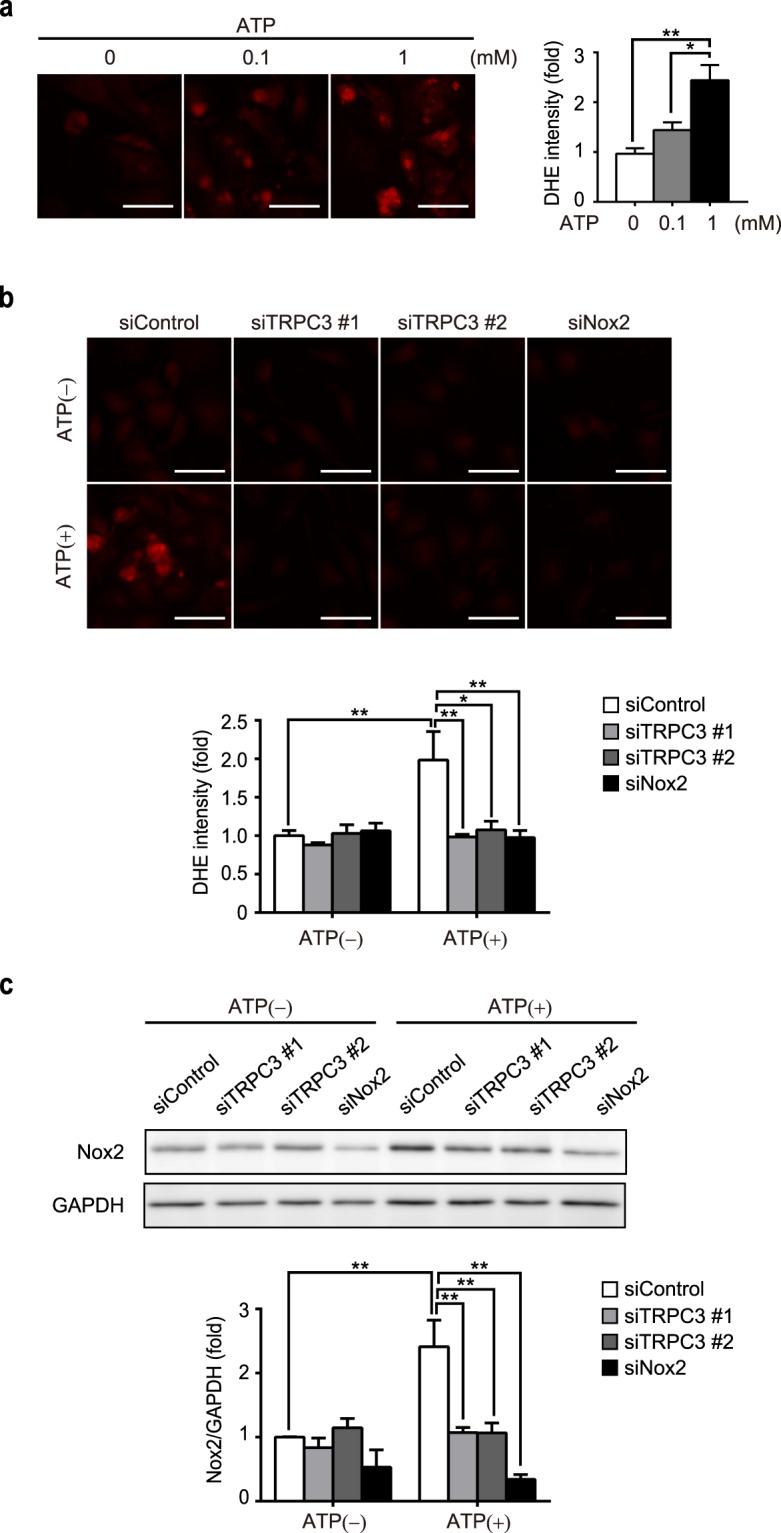
TRPC3 and Nox2 mediate ATP-induced ROS production in NRCMs. (**a**) DHE staining of NRCMs 24 h after ATP treatment (0.1 mM or 1 mM). Fold increase of DHE fluorescence intensity was quantified (n = 3). Scale bars, 50 μm. (**b**) Representative images of DHE staining for siRNA-transfected NRCMs 24 h after ATP treatment (1 mM). Scale bars, 50 μm. n = 3. (**c**) Nox2 protein abundances in NRCMs transfected with siRNAs, following 1 mM ATP treatment for 24 h. n = 3. *P < 0.05; **P < 0.01 (one-way ANOVA; Tukey’s *post hoc* test).

**Figure 3:**
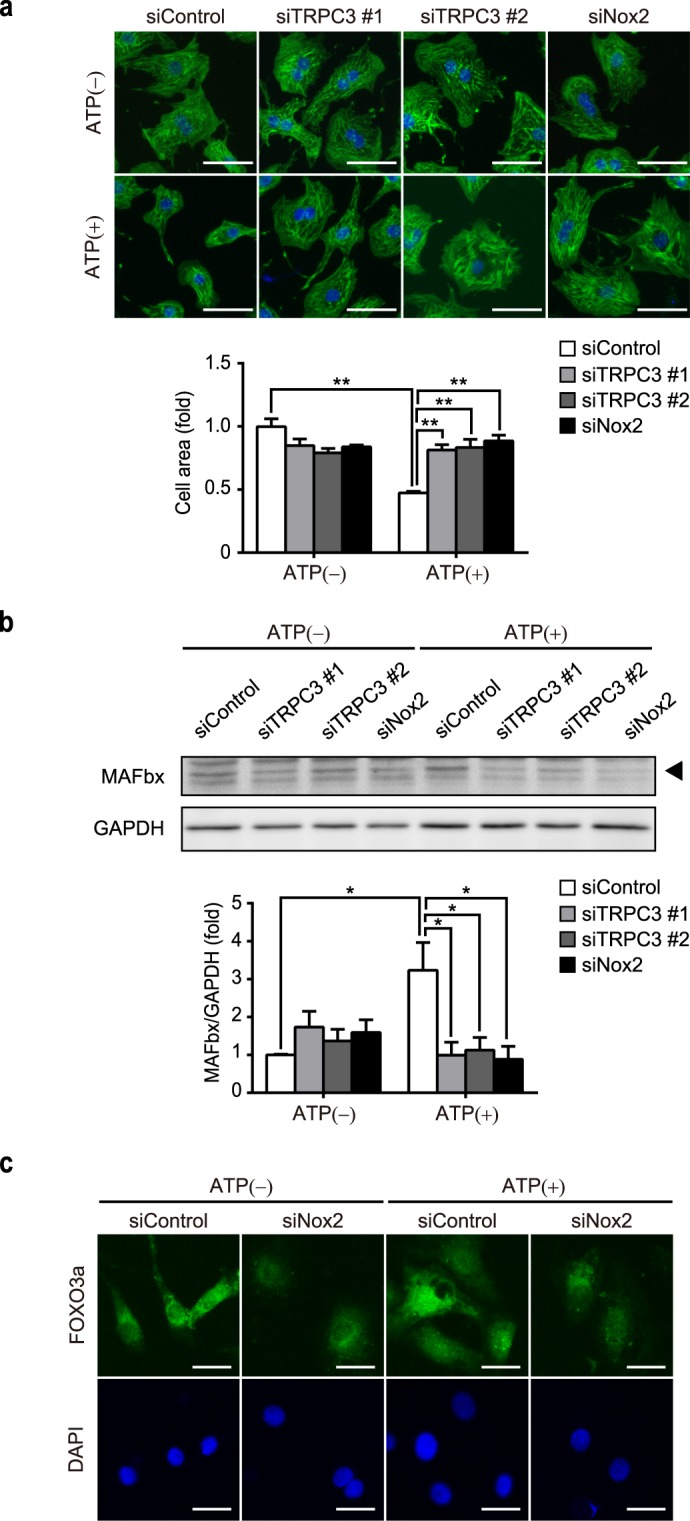
TRPC3-Nox2 coupling mediates ATP-induced atrophy in NRCMs. (**a**) Representative images of phalloidin (green) and DAPI (blue) staining on NRCMs, transfected with siRNA for either TRPC3 or Nox2, following ATP (1 mM) treatment for 24 h. Scale bars, 50 μm. n = 3. **P < 0.01 (one-way ANOVA; Tukey’s *post hoc* test). (**b**) MAFbx protein abundances in NRCMs transfected with siRNAs, with or without treatment of 1 mM ATP for 24 h. n = 3. *p < 0.05 (one-way ANOVA; Tukey’s *post hoc* test). (**c**) Representative images of FoxO3a immunostaining (green) counterstained with DAPI (blue), transfected with siRNA for Nox2, following ATP (1 mM) treatment for 3 h. Scale bars, 20 μm.

ROS have been shown to be involved in muscle cell atrophic response^24^. Using a dihydroethidium (DHE) staining, we found that 1 mM ATP significantly increased the production of ROS (Fig. 2a). We had previously demonstrated that upregulation of Nox2 that forms a stable complex with TRPC3 increased ROS production in doxorubicin-treated cardiomyocytes^19^. Therefore, we examined the effects of TRPC3 and Nox2 gene knockdown in ATP-induced ROS production and Nox2 activation. Introduction of short interfering RNA (siRNA) for TRPC3 and Nox2 significantly attenuated the increase in ROS production, induced by 1 mM ATP (Fig. 2b). In addition, treatment with ATP significantly increased the expression level of Nox2 protein (Fig. 2c). This upregulation of Nox2 was suppressed by gene knockdown of TRPC3 (Fig. 2c). Furthermore, shrinkage of NRCMs caused by ATP treatment was significantly attenuated by the knockdown of either TRPC3 or Nox2 (Fig. 3a). Lastly, we confirmed that ATP-induced atrophy was accompanied by the induction of atrogin-1/MAFbx (Fig. 3b) and nuclear localization of FoxO3a (Fig. 3c), both of which were significantly attenuated by gene knockdown of Nox2 (Fig. 3b,c). Taken together, these results suggest that Nox2 is involved in cardiac atrophy and ROS production induced by ATP.

### Formation of TRPC3-Nox2 complex by P2Y receptor stimulation

**Figure 4:**
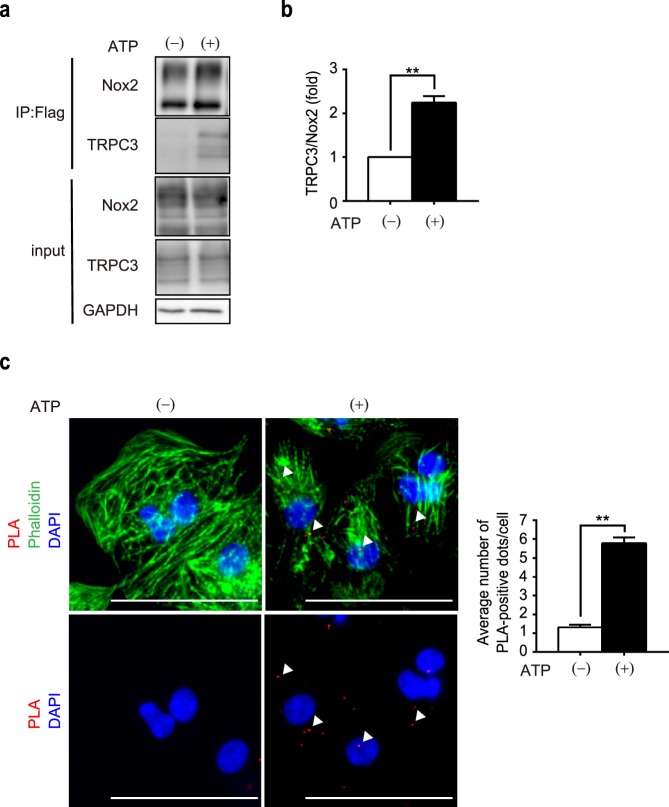
ATP induces interaction between TRPC3 and Nox2. (**a**) Co-immunoprecipitation of Flag-Nox2 with TRPC3-EGFP in HEK293 cells. Cells were stimulated with 1 mM ATP for 3 h. (**b**) Quantification of co-immunoprecipitated TRPC3-EGFP normalized with Flag-Nox2 (n = 3; unpaired *t* test). (**c**) Representative images of TRPC3-Nox2 complexes as measured by Duolink PLA in NRCMs, treated with 1 mM ATP for 12 h. PLA signals are shown as red spots (arrowheads), counterstained with phalloidin (green) and DAPI (blue). Note the cell shrinkage and increased PLA signals by ATP treatment (left). Average number of PLA signals for each cell was quantified (n = 3, right). Scale bars, 50 μm. **P < 0.01 (unpaired *t* test).

We previously showed that interaction between TRPC3 and Nox2 contributes to doxorubicin-induced cardiomyocyte atrophy^19,25^. To examine whether high ATP promotes the formation of TRPC3-Nox2 complex, we used HEK293 cells, which abundantly express endogenous P2Y_1_ and P2Y_2_ receptors. Immunoprecipitation revealed that stimulation of endogenous P2Y receptors by high ATP promoted the interaction of Nox2 and TRPC3 proteins in HEK293 cells without any increase in their protein abundances (Fig. 4a,b). We further verified the interaction and co-localization between endogenous TRPC3 and Nox2 proteins by proximity ligation assay (PLA). PLA signals, representing TRPC3-Nox2 complexes, were found to be more evident in ATP-treated NRCMs compared to non-treated control NRCMs (Fig. 4c). These results suggest that high ATP treatment induces formation of TRPC3-Nox2 protein complexes, likely through P2Y receptor(s) stimulation.

### ATP induces cardiomyocyte atrophy through P2Y_2_ receptor

**Figure 5:**
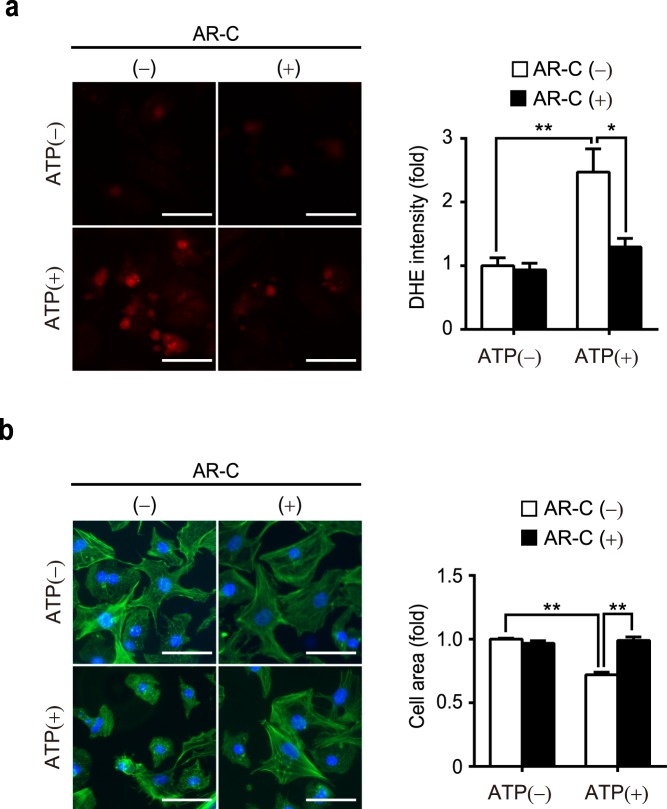
Inhibition of P2Y_2_R attenuates ATP-induced ROS production and atrophy of NRCMs. Effect of P2Y_2_R inhibitor AR-C118925 (AR-C; 10 μM) on ATP-induced ROS production and atrophy in NRCMs, examined by DHE intensity (**a**) and phalloidin staining (green) (**b**), respectively. For phalloidin staining, cell nuclei were counterstained by DAPI (blue). Scale bars, 50 μm. n = 3. *P < 0.05; **P < 0.01 (one-way ANOVA; Tukey’s *post hoc* test).

In the heart, P2Y_2_ receptor (P2Y_2_R) is highly expressed and potently activated by ATP. We had previously demonstrated that ATP activates TRPC5-mediated nitric oxide signaling through P2Y_2_R, whereby suppressing hypertrophic response in cardiomyocytes^18^. In light of these findings, we tested whether ATP induces cardiomyocyte atrophy directly through P2Y_2_R, in addition to its inhibitory effect towards hypertrophy. Treatment of AR-C118925, a specific P2Y_2_R antagonist^26^, significantly reduced ROS production following ATP treatment (Fig. 5a). Moreover, the decrease in cell size was significantly attenuated by AR-C118925 (Fig. 5b). These findings suggest that ATP induces cardiomyocyte atrophy through P2Y_2_R.

### Nutrient deficiency-induced cell atrophy through TRPC3-Nox2 coupling

**Figure 6:**
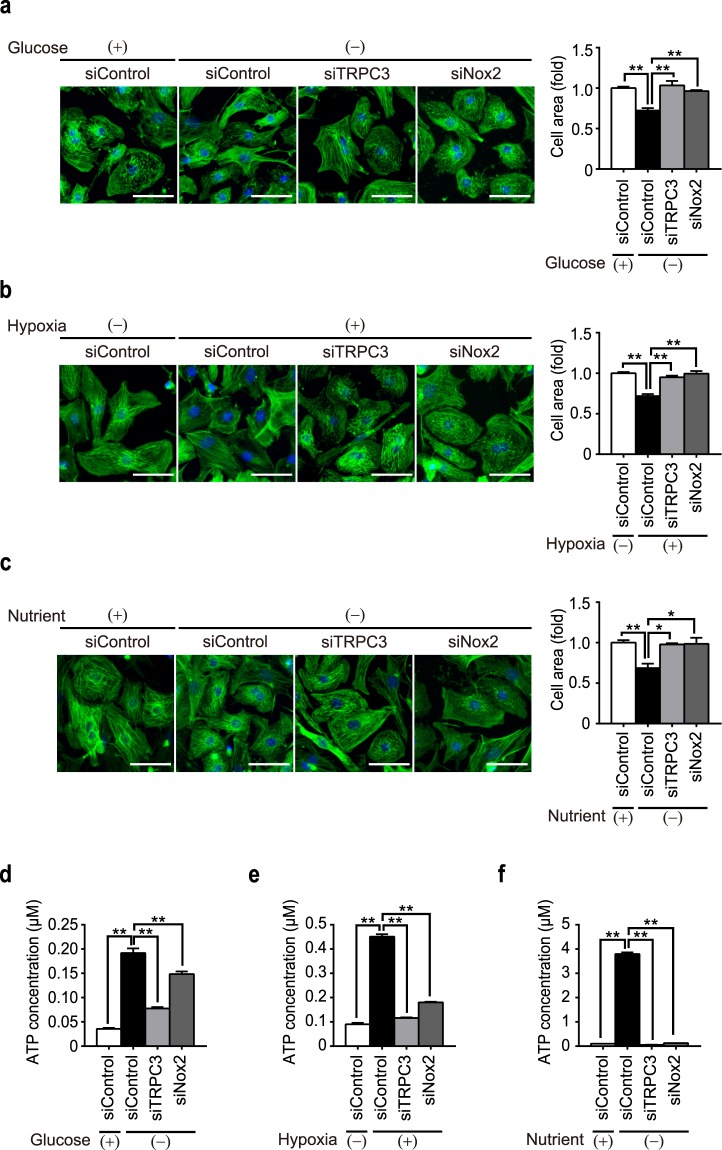
Nutritional deficiency induces cardiomyocyte atrophy and ATP release via TRPC3-Nox2 axis. (**a–c**) Representative images of phalloidin staining on NRCMs treated with glucose deprivation (DMEM without glucose) for 6 h (**a**, n = 3), hypoxia (7% O_2_) for 6 h (**b**, n = 4), and nutrient deprivation (HBSS) for 6 h (**c**, n = 3). Scale bars, 50 μm. (**d–f**) Extracellular ATP concentration in cell culture medium exposed to glucose deprivation (DMEM without glucose) for 6 h (**d**), hypoxia (7% O_2_) for 6 h (**e**), and nutritional deprivation (HBSS) for 6 h (**f**). n = 3. *P < 0.05; **P < 0.01 (one-way ANOVA; Tukey’s *post hoc* test).

Protein turnover are highly regulated by signaling pathways that are triggered by physiological and pathophysiological stresses, such as autophagy induced by nutrient depletion. Therefore, we investigated whether nutrient depletion induces cardiomyocyte atrophy occurring through ATP release and via TRPC3-Nox2 complex formation. To evaluate the effect of pathophysiological stressors on cardiomyocyte atrophy and on TRPC3-Nox2 coupling, glucose starvation, hypoxia and nutrient deprivation were performed on NRCMs. The removal of glucose from the culture media for 6 h resulted in significant reduction in cell size, which was significantly suppressed by siRNAs for TRPC3 and Nox2 as compared to siControl-treated NRCMs (Fig. 6a). Similarly, under 7% hypoxic or nutrient deprived conditions (treated for 6 h), the size of NRCMs were reduced significantly and knockdown of either TRPC3 or Nox2 genes significantly attenuated hypoxia- and nutrient deprivation-induced atrophy (Fig. 6b,c).

**Figure 7:**
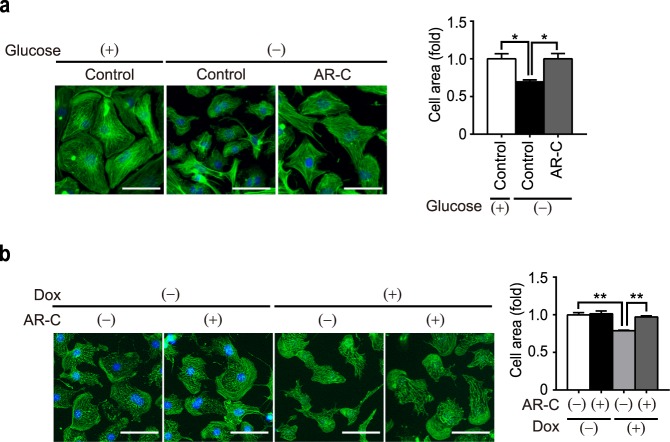
Cardiomyocyte atrophy induced by pathophysiological conditions is attenuated by inhibition of P2Y_2_R. (**a**,**b**) Representative images of phalloidin staining on NRCMs treated with glucose deprivation for 6 h (**a**, n = 3) and doxorubucin (Dox) for 6 h (**b**, n = 3) in the presence of AR-C118925 (AR-C; 10 μM). For all phalloidin staining (green), cell nuclei were counterstained with DAPI (blue). Scale bars, 50 μm. n = 3. *P < 0.05; **P < 0.01 (one-way ANOVA; Tukey’s *post hoc* test).

**Figure 8:**
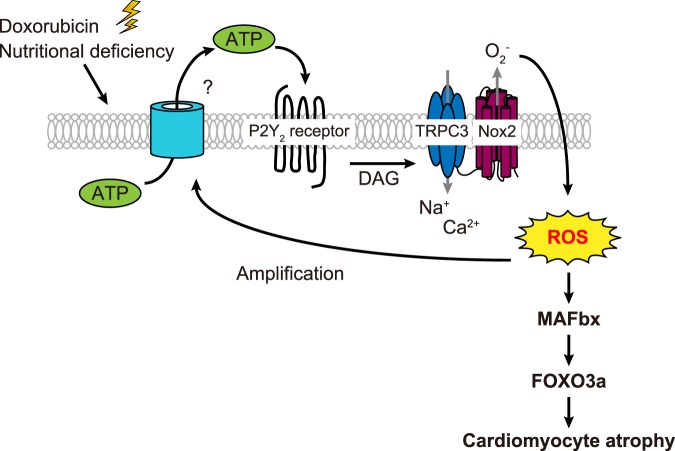
Schematic of the mechanism mediating nutritional deficiency-induced cardiomyocyte atrophy. Pathophysiological stresses such as glucose, oxygen and nutrient deprivation induce extracellular ATP release. The released ATP activates P2Y_2_R at the plasma membrane and induces TRPC3-Nox2 coupling, resulting in overproduction of ROS. Excessive amount of ROS not only triggers atrophy, but also induces further ATP release and amplifies atrophic responses.

Next, we checked if nutrient depletion induces ATP release from NRCMs. To this end, we analyzed the concentration of extracellular ATP in cell culture medium after exposing nutritional stresses. As a result, nutritional stresses such as glucose deprivation, hypoxia, and nutrient deprivation for 6 h, all increased extracellular ATP concentration (Fig. 6d–f), suggesting that NRCMs release ATP in response to nutritional deprivation. To our surprise, knockdown of either TRPC3 or Nox2 significantly suppressed ATP release from stressed NRCMs (Fig. 6d–f), suggesting a positive feedback loop where TRPC3-Nox2 complex further induces the release of ATP. Finally, the application of AR-C118925 significantly attenuated cardiomyocyte atrophy induced by glucose deprivation (Fig. 7a), hypoxia and nutrient deprivation (see Supplementary Fig S2). In addition, AR-C118925 attenuated doxorubicin-induced atrophy (Fig. 7b), which was found to be mediated by TRPC3-Nox2 complex in our previous study^19^. Taken together, these findings suggest that atrophy of NRCMs observed under pathophysiological stresses is triggered by ATP-P2Y_2_R signaling, involving stabilized formation of TRPC3-Nox2 complex, which may promote ROS production (Fig. 8).

## Discussion

In the present study, we demonstrated the atrophic effect of ATP on cardiomyocytes, with little effect on viability even in high (1 mM) concentration (Fig. 1). This atrophy was accompanied by overproduction of ROS (Fig. 2), which was mediated by TRPC3-Nox2 complex formation (Figs 3 and 4). Furthermore, we found that P2Y_2_R contributes to ATP-induced ROS production and atrophy. Lastly, atrophy of cardiomyocyte and extracellular release of ATP were observed in nutritional deficiency, which were dependent on TRPC3 and Nox2 (Figs 6 and 7).

Although extracellular ATP undergoes rapid degradation upon release, several studies have shown that up to millimolar concentration of ATP can be found in response to tissue damage or platelet degranulation^27,28^, which corresponds to the concentration used in the present study. Several pathophysiological stresses that has been related to ATP release and atrophy are ischemic stress involving hypoxia and glucose starvation^29^, nutrient deprivation^30^ and inflammation^31^. We revealed that such nutritional deprivation (glucose, amino acids and oxygen deficiency) causes atrophy on cardiomyocytes *in vitro*. It has been demonstrated that skeletal muscle atrophy in response to fasting or nutrient deprivation involves activation of ubiquitin protease system and other atrophic programs, such as autophagy^32^. In HeLa cells, it has been reported that starvation-induced autophagy is regulated by mitochondria-generated ROS, such as superoxide (O_2_^−^)^33^. It has been also shown in the same cell line that, upon starvation, ATP-containing intracellular vesicles fuse with the plasma membrane and release the nucleotide into the extracellular medium^34^. In cardiomyocytes, it has been reported that glucose deprivation for 6 hours induces oxidative stress and autophagy^35^. Consistently, our findings suggest that ATP release in response to pathophysiological stresses induces cardiomyocyte atrophy through increased ROS production via formation of stable TRPC3-Nox2 protein complex (Fig. 8). In addition, siRNA silencing of either TRPC3 or Nox2 attenuated overproduction of ROS following ATP treatment, indicating the critical role of TRPC3-Nox2 coupling in ATP-induced cardiomyocyte atrophy. Therefore, stabilization of Nox2, an abundant ROS-generating enzyme in cardiomyocytes, appears to be a major source of ROS under ATP-releasing stresses. However, the molecular entity of ROS-mediated ATP release still remains largely obscure. One plausible candidate is pannexin-1 hemi-channel, a redox-sensitive ATP-release channel that is involved in mechanical stress-induced cardiac fibrosis^36^. Moreover, we found that knockdown of either TRPC3 or Nox2 significantly suppressed ATP release from stressed NRCMs (Fig. 6d–f), suggesting a positive feedback loop where TRPC3-Nox2 complex further induces the release of ATP (Fig. 8). This mechanism might be the plausible explanation for sustained release of ATP under cellular stresses, inducing cardiomyocyte atrophy as well as single-dose administration of high ATP (Figs 1–5). Further studies are necessary to elucidate the molecular details of ATP release driven by ROS production via TRPC3-Nox2 axis.

The balance between muscle atrophy and hypertrophy are precisely regulated in response to various stimuli: muscle atrophy is a debilitating response to starvation, immobilization, cancer, and other systemic diseases^5^, whereas muscle hypertrophy is associated with mechanical stress, neurohumoral factors^37^ and ageing^38^. Our data suggest that cardiomyocyte atrophy is triggered by TRPC3-Nox2 functional coupling through P2Y_2_R stimulation. Furthermore, our findings suggest that atrophy induced by fasting or nutrient deprivation is also mediated by P2Y_2_R (Fig. 7). On the other hand, our previous study has shown that ATP induces production of nitric oxide (NO), which negatively regulates hypertrophic signaling mediated by TRPC3/6 channels in cardiomyocytes^18^. Although molecular details of how P2Y_2_R stimulation increases physical interaction between TRPC3 and Nox2 is still unclear, these results suggest that ATP plays key role in mediating cardiac atrophy through P2Y_2_R, as well as inhibiting hypertrophic response.

As atrophic remodeling of the heart in patients with cancer can be a result of both the disease itself and various cancer therapies, limiting the cardiac damage has become an increasingly important issue for the elevation of survival rate in patients during the recovery period. We previously reported that doxorubicin causes myocardial atrophy in mice through overproduction of ROS by upregulated Nox2^19^. Our previous findings also revealed the functional role of TRPC3 as a positive regulator of ROS by forming a stable protein complex with Nox2 in pathophysiological conditions *in vivo*^20,39,40^. Here, we demonstrated that doxorubicin-induced cardiomyocyte atrophy was attenuated by P2Y_2_R inhibitor (Fig. 7b). Taken together, it is likely that P2Y_2_R is the upstream mediator of ROS overproduction involved in doxorubicin-induced cardiomyocyte atrophy, as well as other stressor-mediated atrophy.

Limitations of study: our current study uncovers a new pathway in the development of cardiomyocyte atrophy, where TRPC3-Nox2 axis, activated by extracellular ATP, mainly contributes to nutritional deficiency-induced atrophy through ROS production. However, this study is limited to NRCMs and does not provide direct evidence *in vivo*. Future studies incorporating human disease models (involving cardiac and skeletal muscle atrophy) and knockout approaches can address this question. Furthermore, translating these discoveries to human cardiac atrophy, muscular dystrophy, sarcopenia and cachexia is an overarching future challenge.

## Methods

### Animals

All experiments using animals were approved by an Ethics Committee of National Institutes of Natural Sciences and carried out in accordance with their guidelines. Sprague-Dawley (SD) rats were purchased from Japan SLC Inc.

### Isolation of NRCM and cell culture

NRCMs were prepared from the ventricles of 1–2-day-old SD rats as described previously^41^. Overnight pre-digestion of left ventricular tissue were done using 0.05% trypsin-EDTA (Gibco) at 4 °C, followed by tissue digestion using 1 mg ml^−1^ collagenase type 2 (Worthington) in PBS for 30 min at 37 °C. The dissociated cells were then plated in a 10-cm culture dish containing DMEM supplemented with 10% FBS and 1% penicillin and streptomycin and incubated at 37 °C in a humidified atmosphere (5% CO_2_, 95% air) for 1 h. Floating NRCMs were collected and plated into matrigel (Corning)-coated culture dishes at a density of around 1.5 × 10^5^ cells/ml for 12 well plate and 60 mm dish or 3 × 10^5^ cells/ml for 96 well plate. After 24 h, the culture medium was changed to serum-free DMEM supplemented with 5 mM taurine, 1% penicillin and streptomycin, incubated for more than 2 days before the experiment. For knockdown study, the cells were transfected with siRNAs (20 nM) for 72 h using Lipofectamine RNAiMAX (Thermo Fisher scientific). Stealth siRNAs for rat TRPC3 (#1, RSS329520; #2, RSS329521) and Nox2 (RSS330363) were from Invitrogen. To expose NRCMs with physiological stresses, cells were cultured in DMEM without serum and glucose (glucose deprivation), 7% O_2_ (hypoxia) in a multigas incubator (Panasonic), or Hepes-buffered saline solution (HBSS: 10 mM Hepes (pH 7.4), 140 mM NaCl, 5.6 mM KCl, 10 mM glucose, 1.2 mM CaCl_2_ and 1 mM MgCl_2_; Nutrition deprivation). For the control of nutrition deprivation, mixture of aminoacids (amino acids Mixture standard solution, type H, Wako) were added to HBSS. A selective and competitive P2Y_2_R antagonist (AR-C118925, Tocris, 4890) was reconstituted to 10 mM in DMSO and used at a 10 μM working dilution. For plasmid transfection, HEK293 cells were cultured in DMEM (10% FBS and 1% penicillin and streptomycin) and transfected with X-tremeGENE9 (Roche) according to manufacturer’s instruction.

### Cell viability assays

LDH assay and cell counting assay were performed using Cytotoxicity LDH Assay Kit-WST and Cell Counting Kit-8 (Dojindo) respectively. Briefly, NRCMs were seeded in 96-well plate with serum free media for 48 h before ATP treatment (1 mM). NRCMs were further incubated with LDH assay and cell counting reagents respectively and measured using Spectra Max i3 (Molecular device) following the manufacturer’s instruction.

### Measurements of cell size and ROS production in NRCM

Non-transfected or siRNA-transfected NRCMs were fixed in 2% or 4% paraformaldehyde (PFA) for ROS production and cell size analysis, respectively. For cell size analysis, NRCMs were permeabilized with 0.5% Triton X-100 in Tris-buffered saline with Tween 20 (0.5% TBST) and blocked with 2% bovine serum albumin in 0.1% TBST. NRCMs were then stained with Alexa Fluor 488-conjugated Phalloidin (Thermo Fisher Scientific), and cell surface areas were observed using a fluorescence microscope and analyzed in at least 50 cells in each experiment using ImageJ software. Productions of ROS in NRCMs were measured using DHE. DHE staining for non-transfected or siRNA-transfected NRCMs were performed by the incubation with DHE (2 μM) for 1 h. Then, the averaged DHE fluorescence intensity was quantified using BZ-II Analyzer software (Keyence), by averaging intensity of 40–50 cells in each experiment.

### Western blot and immunoprecipitation

Non-transfected or siRNA-transfected NRCMs were harvested in lysis buffer containing 0.1% SDS, 0.5% sodium deoxycholate, 140 mM NaCl, 20 mM Tris-HCl (pH 7.4), 2 mM EDTA, 1% Triton X-100, 10% glycerol and protease inhibitor cocktail (Nacalai). Protein lysates (10 μg) were separated by SDS-PAGE and transferred onto PVDF membranes (Millipore). The desired proteins were then detected using primary antibodies against Nox2 (1:2,000; sc-130543), atrogin/MAFbx (1:2000; sc-166806) and GAPDH (1:2,000; sc-25778) from Santa Cruz Biotechnology Inc. Membranes were then incubated with the secondary antibodies; anti-rabbit IgG-HRP (7074) and anti-mouse IgG-HRP (7076) from Cell Signaling Technology. Immunoreactive bands were detected using Western Lightning Plus ECL (PerkinElmer) and images were captured with an ImageQuant LAS 4000 (GE healthcare Life Science). The bands intensity was quantified using ImageQuant TCL software (GE Healthcare Life Sciences). To analyze Nox2 and TRPC3 interaction, protein complex was concentrated by co-immunoprecipitation (co-IP). Briefly, cell lysates from ATP-treated transfected-HEK293 cells were harvested in IP buffer containing 50 mM HEPES, 150 mM NaCl, 50 mM NaF, 1.5 mM MgCl_2_, 1 mM EDTA, 10% glycerol, 1% Triton-X 100 and protease inhibitor cocktail (Thermo Fisher Scientific). Flag-tagged protein complex was immunoprecipitated using bed volume of anti-flag M2 Affinity Gel (Sigma Aldrich). After incubation for 24 h, the immune complexes was washed 3 times with IP buffer, suspended in SDS sample buffer containing 0.1 M DTT, and incubated for 1 h at room temperature. TRPC3 and Nox2 was detected with rabbit anti-GFP antibody (1:1,000, Cell Signaling #2956) and mouse anti-Nox2 (1:2,000, sc-130543).

### Immunostaining

For evaluating subcellular distribution of FoxO3a, NRCMs were permeabilized with 0.5% Triton X-100 in Tris-buffered saline with Tween 20 (0.5% TBST) and blocked with 2% bovine serum albumin in 0.1% TBST. NRCMs were then incubated with anti-FoxO3a antibody (1:100; Cell Signaling #12829) for 2 h at room temperature. After washing with PBS, cells were incubated with Alexa Fluor 488-conjugated anti-Rabbit secondary antibody (Thermo Fisher Scientific) for 1 h at room temperature. Then, cells were mounted with ProLong™ Diamond Antifade Mountant with DAPI (Thermo Fisher Scientific). Images were obtained using BZ-X700 fluorescence microscope (Keyence).

### Proximity ligation assay (PLA)

To determine TRPC3-Nox2 interaction in NRCMs, proximity ligation assay was conducted using Duolink PLA Fluorescence (Sigma Aldrich) according to the manufacturer’s instruction. After fixing and blocking, NRCMs were incubated with rabbit anti-TRPC3 (Alomone lab) and mouse anti-Nox2 (Santa Cruz) followed by 1 h PLA probes incubation. The ligation (30 min) and amplification (100 min) steps were performed in 37 °C chamber and NRCMs were nuclear stained with DAPI. Images were captured using a fluorescence microscope (BZ-X710, Keyence) and the number of PLA signal-positive puncta were quantified and analyzed using ImageJ (National Institutes of Health) software.

### Extracellular ATP assay

After incubating in serum-free DMEM for more than two days, non-transfected and transfected NRCMs were placed under glucose starvation (DMEM without glucose) for 6 h, hypoxia (7% O_2_) for 6 h and nutrient deprivation (HBSS without amino acids supplemented with CaCl_2_ and MgCl_2_) for 6 h. Extracellular ATP concentration present in the medium were snapped frozen and stored in −80 °C before measured using ATP Bioluminescence Assay kit CLSII (Roche) according to a manufacturer’s instruction.

### Statistical analysis

All results were presented as the mean ± SEM from at least 3 independent experiments. Statistical comparisons were made using unpaired *t* tests for two-group comparisons or using one-way ANOVA followed by Tukey’s *post hoc* test for multiple groups. Values of P < 0.05 were considered to be statistically significant.

## Supplementary information

Supplementary Dataset 1

## References

[1] Hill JA, Olson EN (2008). Cardiac Plasticity. N. Engl. J. Med..

[2] Sengupta A, Molkentin JD, Yutzey KE (2009). FoxO transcription factors promote autophagy in cardiomyocytes. J. Biol. Chem..

[3] Cao DJ (2013). Mechanical unloading activates FoxO3 to trigger Bnip3-dependent cardiomyocyte atrophy. J. Am. Heart Assoc..

[4] Matsui Y (2007). Distinct roles of autophagy in the heart during ischemia and reperfusion: roles of AMP-activated protein kinase and Beclin 1 in mediating autophagy. Circ. Res..

[5] Sandri M (2004). Foxo transcription factors induce the atrophy-related ubiquitin ligase atrogin-1 and cause skeletal muscle atrophy. Cell.

[6] Skurk C (2005). The FOXO3a transcription factor regulates cardiac myocyte size downstream of AKT signaling. J. Biol. Chem..

[7] Trautmann A (2009). Extracellular ATP in the immune system: More than just a ‘danger signal’. Sci. Signal..

[8] Wang Y, Roman R, Lidofsky SD, Fitz JG (1996). Autocrine signaling through ATP release represents a novel mechanism for cell volume regulation. Proc. Natl. Acad. Sci. USA.

[9] Hopwood AM, Lincoln K, Kirkpatrick J, Burnstock KA, Adenosine G (1989). 5′-triphosphate, adenosine and endothelium-derived relaxing factor in hypoxic vasodilatation of the heart. Eur. J. Pharmacol..

[10] Ralevic V, Burnstock G (1991). Roles of P2-purinoceptors in the cardiovascular system. Circulation.

[11] Ralevic V (2001). Mechanism of prolonged vasorelaxation to ATP in the rat isolated mesenteric arterial bed. Br. J. Pharmacol..

[12] Yegutkin GG (1783). Nucleotide- and nucleoside-converting ectoenzymes: Important modulators of purinergic signalling cascade. Biochim. Biophys. Acta.

[13] Balla T (2009). Regulation of Ca2+ entry by inositol lipids in mammalian cells by multiple mechanisms. Cell Calcium.

[14] Numaga T (2010). Ca2+ influx and protein scaffolding via TRPC3 sustain PKCbeta and ERK activation in B cells. J. Cell Sci..

[15] Patterson RL, Boehning D, Snyder SH (2004). Inositol 1,4,5-trisphosphate receptors as signal integrators. Annu. Rev. Biochem..

[16] Post GR, Goldstein D, Thuerauf DJ, Glembotski CC, Brown JH (1996). Dissociation of p44 and p42 mitogen-activated protein kinase activation from receptor-induced hypertrophy in neonatal rat ventricular myocytes. J. Biol. Chem..

[17] Zheng JS, Boluyt MO, O’Neill L, Crow MT, Lakatta EG (1994). Extracellular ATP induces immediate-early gene expression but not cellular hypertrophy in neonatal cardiac myocytes. Circ. Res..

[18] Sunggip C (2018). TRPC5-eNOS axis negatively regulates ATP-induced cardiomyocyte hypertrophy. Front. Pharmacol..

[19] Shimauchi T (2017). TRPC3-Nox2 complex mediates doxorubicin-induced myocardial atrophy. JCI Insight.

[20] Kitajima N (2016). TRPC3 positively regulates reactive oxygen species driving maladaptive cardiac remodeling. Sci. Rep..

[21] Qiu J (2018). Mechanistic Role of Reactive Oxygen Species and Therapeutic Potential of Antioxidants in Denervation- or Fasting-Induced Skeletal Muscle Atrophy. Front. Physiol..

[22] Powers SK, Duarte J, Kavazis AN, Talbert EE (2010). Reactive oxygen species are signalling molecules for skeletal muscle adaptation. Experimental Physiology.

[23] Powers SK, Smuder AJ, Criswell DS (2011). Mechanistic links between oxidative stress and disuse muscle atrophy. Antioxid. Redox Signal..

[24] Powers, S. K., Morton, A. B., Ahn, B. & Smuder, A. J. Redox control of skeletal muscle atrophy, 208–217, 10.1016/j.freeradbiomed.2016.02.021.Redox (2017). 10.1016/j.freeradbiomed.2016.02.021 PMC5006677 26912035

[25] Kitajima, N., Numaga-tomita, T., Watanabe, M. & Kuroda, T. TRPC3 positively regulates reactive oxygen species driving maladaptive cardiac remodeling, 1–14, 10.1038/srep37001 (2016). 10.1038/srep37001 PMC5105134 27833156

[26] Rafehi M, Burbiel JC, Attah IY, Abdelrahman A, Muller CE (2017). Synthesis, characterization, and *in vitro* evaluation of the selective P2Y2 receptor antagonist AR-C118925. Purinergic Signal..

[27] Gordon JL (1986). Extracellular ATP: effects, sources and fate. Biochem. J..

[28] Pellegatti P (2008). Increased level of extracellular ATP at tumor sites: *In vivo* imaging with plasma membrane luciferase. PLoS One.

[29] Faigle M, Seessle J, Zug S, El Kasmi KC, Eltzschig HK (2008). ATP release from vascular endothelia occurs across Cx43 hemichannels and is attenuated during hypoxia. PLoS One.

[30] Paul PK (2012). The E3 ubiquitin ligase TRAF6 intercedes in starvation-induced skeletal muscle atrophy through multiple mechanisms. Mol. Cell. Biol..

[31] Dolmatova E (2012). Cardiomyocyte ATP release through pannexin 1 aids in early fibroblast activation. Am. J. Physiol. Heart Circ. Physiol..

[32] Lecker SH (2004). Multiple types of skeletal muscle atrophy involve a common program of changes in gene expression. FASEB J. Off. Publ. Fed. Am. Soc. Exp. Biol..

[33] Li L, Chen Y, Gibson SB (2013). Starvation-induced autophagy is regulated by mitochondrial reactive oxygen species leading to AMPK activation. Cell. Signal..

[34] Scherz-Shouval R (2007). Reactive oxygen species are essential for autophagy and specifically regulate the activity of Atg4. EMBO J..

[35] Marambio P (2010). Glucose deprivation causes oxidative stress and stimulates aggresome formation and autophagy in cultured cardiac myocytes. Biochim. Biophys. Acta.

[36] Nishida M (2008). P2Y6receptor-Gα12/13signalling in cardiomyocytes triggers pressure overload-induced cardiac fibrosis. EMBO J..

[37] Heineke J, Molkentin JD (2006). Regulation of cardiac hypertrophy by intracellular signalling pathways. Nature Reviews Molecular Cell Biology.

[38] Dai D-F, Chen T, Johnson SC, Szeto H, Rabinovitch PS (2012). Cardiac Aging: From Molecular Mechanisms to Significance in Human Health and Disease. Antioxid. Redox Signal..

[39] Kitajima N (2011). TRPC3-mediated Ca2+ influx contributes to Rac1-mediated production of reactive oxygen species in MLP-deficient mouse hearts. Biochem. Biophys. Res. Commun..

[40] Numaga-Tomita T (2016). TRPC3-GEF-H1 axis mediates pressure overload-induced cardiac fibrosis. Sci. Rep..

[41] Nishimura A (2018). Hypoxia-induced interaction of filamin with Drp1 causes mitochondrial hyperfission-associated myocardial senescence. Sci. Signal..

