# Supplementary material for: TRPC3-Nox2 axis mediates nutritional deficiency-induced cardiomyocyte atrophy
Source: Sci Rep. 2019 Jul 5;9:9785. doi: 10.1038/s41598-019-46252-2 (PMC6611789; doi:10.1038/s41598-019-46252-2)
Supplement: Supplementary file 1 — Supplementary Dataset 1 [file 41598_2019_46252_MOESM1_ESM.pdf]

Supplementary Information for

TRPC3-Nox2 axis mediates nutritional deficiency-induced cardiomyocyte atrophy

Suhaini Binti Sudi, Tomohiro Tanaka, Sayaka Oda, Kazuhiro Nishiyama ,Akiyuki Nishimura, Caroline Sunggip, Supachoke Mangmool, Takuro Numaga-Tomita and Motohiro Nishida

a

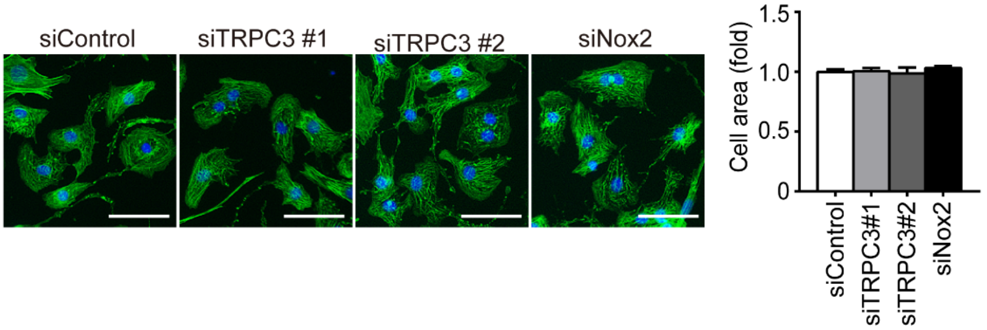

siControl
siTRPC3 #1
siTRPC3 #2
siNox2
Cell area (fold)
1.5
1.0
0.5
0
siControl
siTRPC3#1
siTRPC3#2
siNox2

b

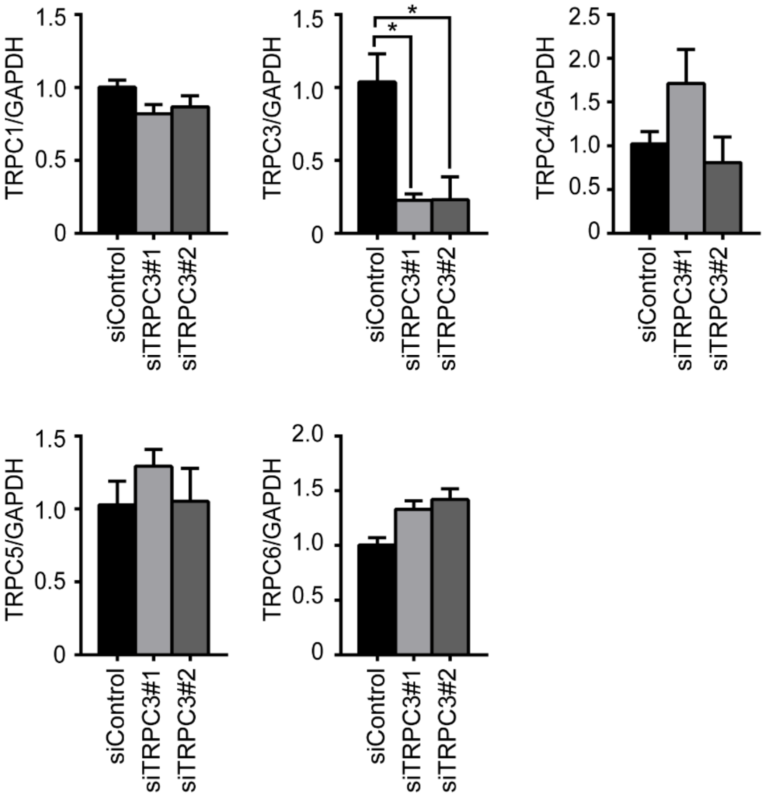

TRPC1/GAPDH
TRPC3/GAPDH
TRPC4/GAPDH
TRPC5/GAPDH
TRPC6/GAPDH
*
*
siControl
siTRPC3#1
siTRPC3#2

c

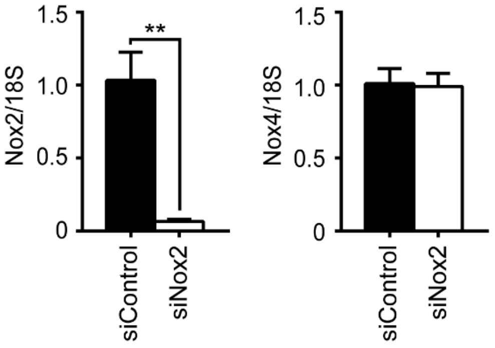

Nox2/18S
Nox4/18S
**
siControl
siNox2

Supplementary Figure S1 Effect of siRNA knockdown for TRPC3 and Nox2. (**a**) Representative images of phalloidin (green) and DAPI (blue) on NRCMs that are transfected with siRNA for TRPC3 (#1, #2) and Nox2. Scale bars, 50 μm. Fold increase of averaged cell area, showing no statistically significant difference between groups (right; n=3; one-way ANOVA; Tukey's *post hoc* test). (**b,c**) Relative mRNA expression level of TRPC isoforms (**b**) and Nox isoforms (**c**) expressed in NRCM, following siRNA transfection for TRPC3 and Nox2, respectively. n=3. *P<0.05; **P<0.01 (one-way ANOVA; Tukey's *post hoc* test)

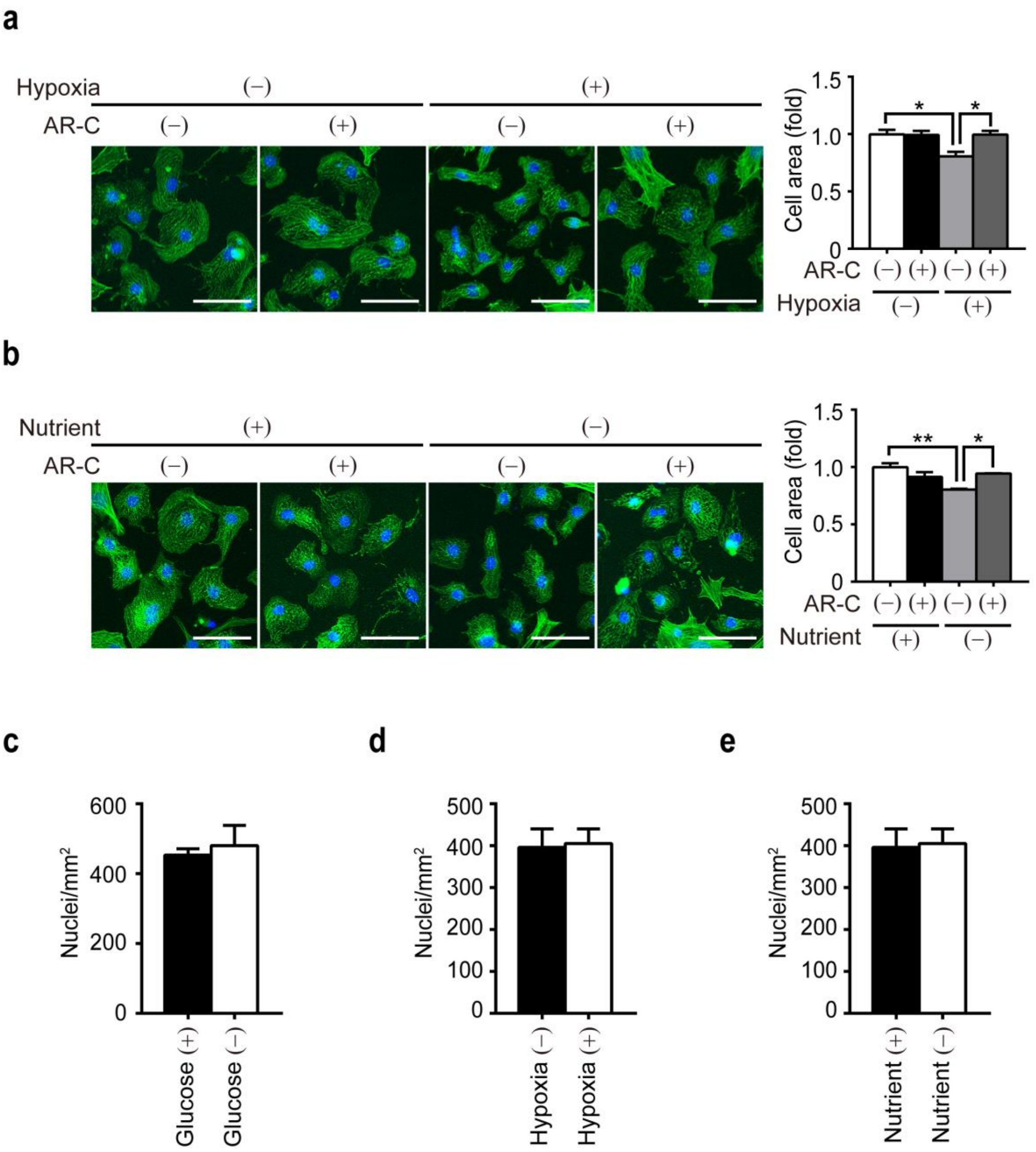

a
Hypoxia
(−)
(+)
AR-C
(−)
(+)
(−)
(+)
Cell area (fold)
1.5
1.0
0.5
0
*
*
AR-C (−) (+) (−) (+)
Hypoxia (−) (+)
b
Nutrient
(+)
(−)
AR-C
(−)
(+)
(−)
(+)
Cell area (fold)
1.5
1.0
0.5
0
**
*
AR-C (−) (+) (−) (+)
Nutrient (+) (−)
c
Nuclei/mm²
600
400
200
0
Glucose (+)
Glucose (−)
d
Nuclei/mm²
500
400
300
200
100
0
Hypoxia (−)
Hypoxia (+)
e
Nuclei/mm²
500
400
300
200
100
0
Nutrient (+)
Nutrient (−)

Supplementary Figure S2 Cardiomyocyte atrophy induced by pathophysiological conditions is attenuated by inhibition of P2Y$_2$R. (**a, b**) Representative images of phalloidin staining on NRCMs treated with hypoxia (7 % O_2) for 6h (**a**), and nutrient deprivation (HBSS) for 6h (**b**), in the presence of AR-C118925 (AR-C; 10 μM). For all phalloidin staining (green), cell nuclei were counterstained with DAPI (blue). Scale bars, 50 μm. n=3. *P<0.05; **P<0.01 (one-way ANOVA; Tukey's *post hoc* test). (**c-e**) Quantitative analysis showing the number of DAPI-positive nuclei per area (mm^2) under glucose deprivation (DMEM without glucose) for 6h (**c**), hypoxia (7 % O_2) for 6h (**d**), and nutrient deprivation (HBSS) for 6h (**e**).

a

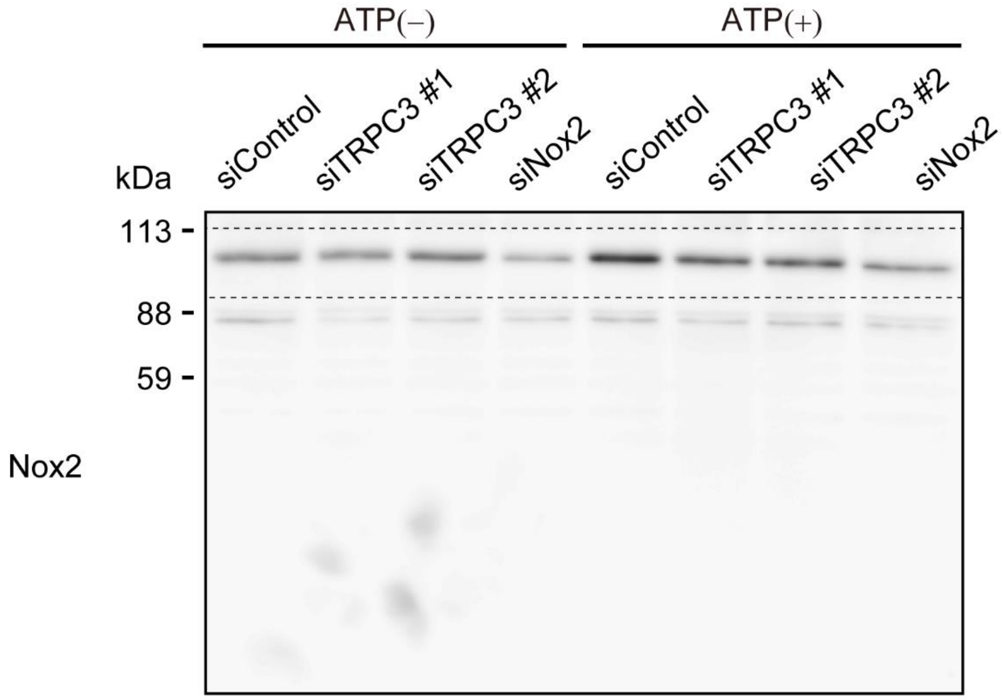

Figure 2c

b

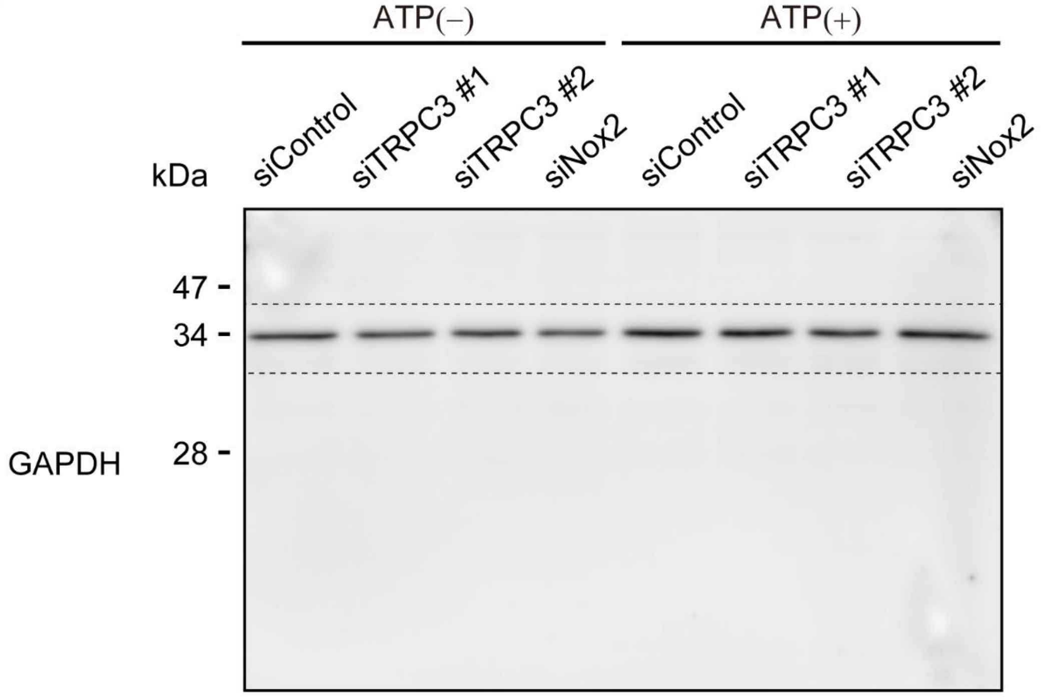

Figure 2c

Supplementary Figure S3. (**a**) Full-length blots in Fig 2c Nox2. (**b**) Full-length blots in Fig 2c GAPDH.

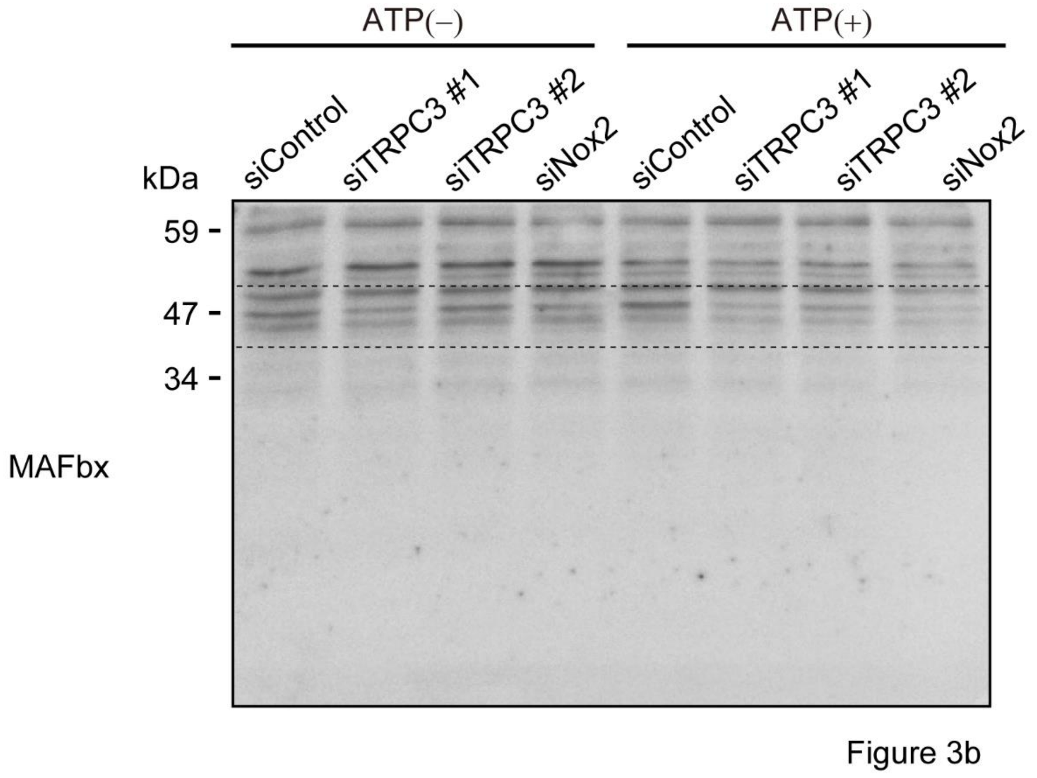

Supplementary Figure S4. Full-length blots in Fig 3b MAFbx. GAPDH blots in Figure 3b are the same as in Figure 2c.

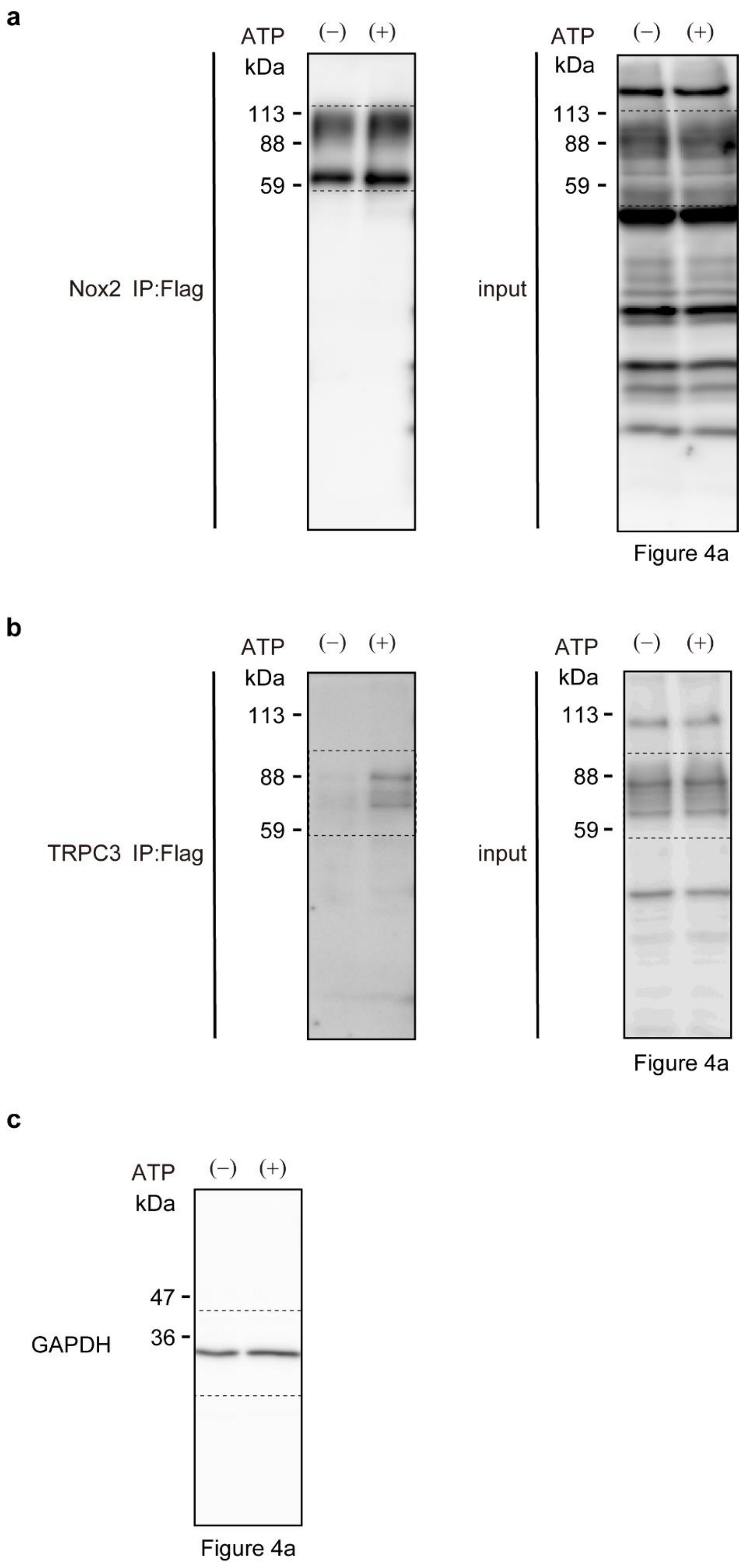
a
ATP (−) (+)
kDa
113
88
59
Nox2 IP:Flag
ATP (−) (+)
kDa
113
88
59
input
Figure 4a
b
ATP (−) (+)
kDa
113
88
59
TRPC3 IP:Flag
ATP (−) (+)
kDa
113
88
59
input
Figure 4a
c
ATP (−) (+)
kDa
47
36
GAPDH
Figure 4a

Supplementary Figure 5. (**a**) Full-length blots in Fig 4a Nox2. (**b**) Full-length blots in Fig 4a TRPC3. (**c**) Full-length blots in Fig 4a GAPDH.

Supplementary Table S1 List of primer sets for real-time PCR.

Gene symbol	Forward primer	Reverse primer
TRPC1	AGGTGAAGGAGGAGAACACCTTG	CCATAAGTTTCTGACAACCGTAGTCC
TRPC3	TGACTTCTGTTGTGCTCAAATATG	CCTTCTGAAGTCTTCTCCTCCTGC
TRPC4	AATTACTCGTCAACAGGCGGC	CACCACCACCTTCTCCGACTT
TRPC5	AAGTTTCGAATTTGAGGAGCAGATG	AATCTCTGATGGCATCGCACA
TRPC6	TCACTTGGAAGAACAGTGAAAGA	CATCCTCAATTTCCTGGAATGAAC

Supplementary Methods

Real-time reverse transcription polymerase chain reaction

Total RNA was isolated from NRCMs using a ReliaPrep™ miRNA Cell and Tissue Miniprep System (Promega) according to the manufacturer's instructions. Complementary DNAs were synthesized using a ReverTra Ace® qPCR RTMaster Mix (Toyobo). Quantitative PCR was performed using KAPA SYBR FAST qPCR master mix (KAPA biosystems) for TRPC isoforms and with Quantitect probe RT-PCR mix (Qiagen) for Nox isoforms, respectively, by LightCycler® 96 System (Roche) according to the manufacturer's instructions. Relative quantification was carried out with reference to GAPDH or 18S ribosomal RNA for TRPCs or Noxs, respectively, and analyzed using the comparative CT method. The primer mix for rodent GAPDH and Taqman probe mixes for Cybb (Nox2), Nox4, and eukaryotic 18S ribosomal RNA were purchased from Applied Biosystems. The primers are listed in Supplementary Table S1.
